# Effect of *Helicobacter pylori* infection on malignancy of undifferentiated-type gastric cancer

**DOI:** 10.1186/s12876-021-02034-7

**Published:** 2022-01-06

**Authors:** Masami Tanaka, Shu Hoteya, Daisuke Kikuchi, Kosuke Nomura, Yorinari Ochiai, Takayuki Okamura, Junnosuke Hayasaka, Yugo Suzuki, Yutaka Mitsunaga, Nobuhiro Dan, Hiroyuki Odagiri, Satoshi Yamashita, Akira Matsui

**Affiliations:** Department of Gastroenterology, Toranomon Hospital, 2-2-2 Toranomon, Minato-ku, Tokyo, 105-8470 Japan

**Keywords:** *Helicobacter pylori*, Undifferentiated carcinoma, Gastric cancer, Malignancy

## Abstract

**Background:**

Although almost all cases of gastric cancer are caused by *Helicobacter pylori* (HP) infection, there are some rare exceptions. Furthermore, the clinicopathological characteristics of gastric cancer may differ depending on HP infection status. This study aimed to determine the clinicopathological characteristics of undifferentiated-type gastric cancer (UD-GC) according to HP status.

**Methods:**

The study involved 83 patients with UD-GC who were selected from 1559 patients with gastric cancer who underwent endoscopic resection at our hospital and whose HP infection status was confirmed. Clinicopathological characteristics were evaluated according to HP status (eradicated, n = 28; infected, n = 32; not infected, n = 23).

**Results:**

In patients without HP infection, UD-GCs were < 20 mm and intramucosal with no vascular invasion. In patients with eradicated HP, there was no correlation between development of UD-GC and time since eradication. Nine of twelve patients with a tumor detected ≥ 5 years after eradication had undergone yearly endoscopy. Submucosal invasion was observed in two of four patients and lymphovascular invasion in three of four patients whose UD-GC was detected ≥ 10 years after eradication. There was no significant between-group difference in the frequency of lesions with invasion into the submucosal layer or deeper (14.3%, 10.5%, and 0% in the UD-E, UD-I, and UD-U groups, respectively).

**Conclusion:**

The clinicopathological characteristics of UD-GC were similar between HP-infected patients and HP-eradicated patients. Three of four patients with eradicated HP whose UD-GC developed ≥ 10 years after eradication were not eligible for endoscopic treatment and required additional surgery resection. In contrast, UD-GC was curable by endoscopic resection in all patients without HP infection.

## Background

Almost all cases of gastric cancer are caused by *Helicobacter pylori* (HP) infection [1–3]. Although rare, gastric cancer can also develop in the absence of HP infection [4–6], and the characteristics of gastric cancer without HP infection have become clearer with accumulation of cases [7–10]. The clinicopathological features of gastric cancer differ according to HP infection status. Differentiated-type gastric cancer (D-GC) undergoes morphological changes upon eradication of HP [11–13], and changes on the mucosal surface of the tumor to a non-cancerous appearance after eradication hinder endoscopic diagnosis [14]. Meanwhile, the degree of malignancy of undifferentiated-type gastric cancer (UD-GC), including proliferative ability and progression rate, is reported to be higher in patients with HP infection (both post-eradication and current infection cases) than in those without HP infection [15]. However, details of UD-GC post-eradication remain unclear. The latest endoscopic mucosal resection/endoscopic submucosal dissection guidelines state that intramucosal lesions measuring ≤ 20 mm and ulcer-negative UD-GC are absolute indications for endoscopic resection [16]. Therefore, it is important for endoscopists diagnosing and treating UD-GC to have a good knowledge of the clinicopathological features of this tumor according to HP infection status.

In this study, we classified patients with UD-GC according to HP status as a post-eradication (UD-E) group, a current infection (UD-I) group, and a non-infected (UD-U) group with the aim of clarifying the clinicopathological features of each group.

## Methods

**Fig. 1:**
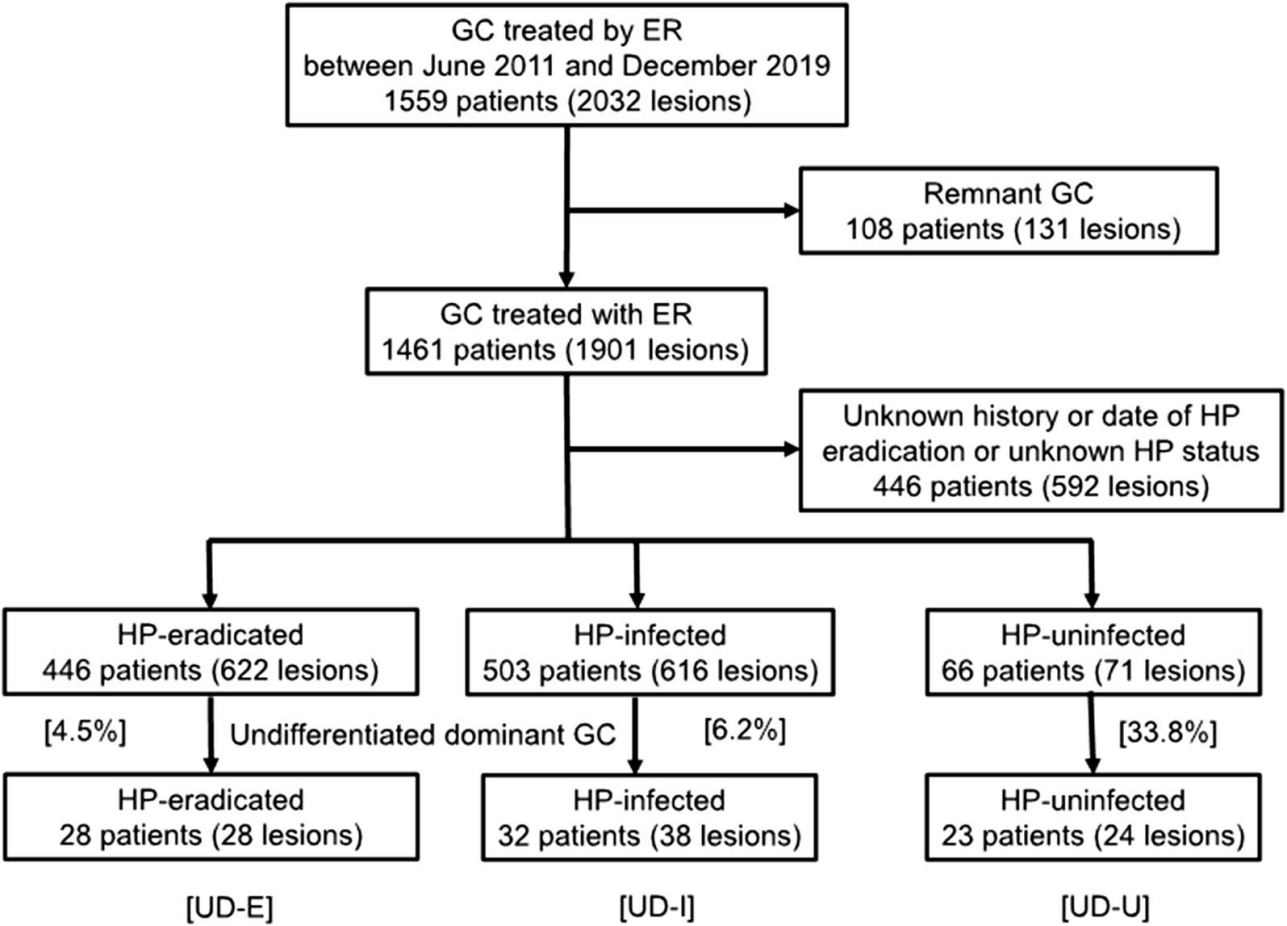
Flow chart showing the patient selection process. Undifferentiated-type cases with confirmed HP infection status were extracted for analysis. GC, gastric cancer; ER, endoscopic resection; HP, *Helicobacter pylori*; UD-E, post-eradication group: UD-I, current infection group; UD-U, uninfected group

The study involved patients with gastric cancer who underwent endoscopic resection at Toranomon Hospital between June 2011 and December 2019. A flow chart showing the patient selection process is provided in Fig. 1. After excluding 108 cases with residual gastric cancer and 446 with unknown HP infection status or unclear time of eradication, we reviewed 1559 cases (2032 lesions) with confirmed HP status. This study was approved by the ethics committee of Toranomon Hospital, approval number 1173.

HP eradication group was defined as follows: known time of eradication and HP-negative status confirmed by a stool antigen test (Meridian Inc., Cincinnati, OH) or a ^13^C-urea breath test (Otsuka Pharmaceutical Co., Ltd., Tokushima, Japan). Current HP infection was defined as a positive urea breath test or stool antigen test, or a positive serum antibody test (E-plate test; Eiken Chemical Co., Ltd., Tokyo, Japan) plus endoscopic findings suggesting current HP infection [17]. Patients were deemed not to have HP infection if the following four criteria were satisfied: no history of HP eradication; a negative serum antibody test (< 3 U/mL) and a negative stool antigen test or urea breath test; no atrophy of the background mucosa on pathological examination of the specimen obtained during endoscopic resection; and no atrophy according to the Kimura-Takemoto classification [18] with regular arrangement of the collecting venules in the lesser curvature of the gastric angle [19].

HP status was confirmed in 446 cases (622 lesions) in HP-eradicated, 503 cases (616 lesions) in HP-infected, and 66 cases (71 lesions) in HP-uninfected. UD-GC cases were then extracted for analysis; there were 28 cases (28 lesions) in the UD-E group, 32 cases (38 lesions) in the UD-I group, and 23 cases (24 lesions) in the UD-U group.

The following clinicopathological characteristics were evaluated: patient demographics (age and sex), endoscopic findings (degree of atrophy, macroscopic type, site, and color tone), and histopathological features (findings, tumor size, depth of invasion, and lymphovascular invasion). In the UD-E group, we also evaluated the time from eradication, tumor size, color tone, depth of invasion, and lymphovascular invasion. The Kimura-Takemoto classification [18] was used to evaluate the degree of atrophy (Close[C]-0, none; C-1 and C-2, mild; C-3 to Open[O]-3), moderate-severe). Color tone was classified as discolored or reddish; if mixed, the predominant tone was selected. Histopathological findings were classified into UD-GC or D-GC; when both types were present, the predominant type was used. UD-GC cases were further divided into pure signet ring cell carcinoma or other type (poorly differentiated adenocarcinoma or mixed poorly differentiated adenocarcinoma and signet ring cell carcinoma) [20].

The clinicopathological features were compared between the three groups using chi square test, Fisher exact test, Kruskal Wallis test, and Scheffe test. All statistical analyses were performed using SPSS version 27 (IBM Corp., Armonk, NY). A *p*-value < 0.05 was considered statistically significant.

## Results

The ratio of UD-GC to all tumors in each HP infection status group was significantly higher in the UD-U group (33.8%) than in the UD-E and UD-I groups (4.5% and 6.2%, respectively), which is shown in Fig. 1.

### Patient characteristics

The characteristics of the patients in the UD-E, UD-I, and UD-U groups are shown in Table 1. The male proportion was 64.3% in the UD-E group, 43.8% in the UD-I group, and 87.0% in the UD-U group. There was a significantly higher proportion of women in the UD-I group than in the UD-U group (*p* = 0.001, UD-I vs UD-U; *p* = 0.105, UD-U vs UD-E). However, there was no significant difference in the sex distribution between the UD-E and UD-I groups or between the UD-E and UD-U groups. The mean age ± standard deviation was 63.1 ± 14.3, 64.0 ± 13.4, and 56.3 ± 9.15 years, respectively, in the UD-E, UD-I, and UD-U groups; patients in the UD-U group were significantly younger than those in the UD-E and UD-I groups (*p* = 0.017 and *p* = 0.019, respectively). The background mucosa was significantly more atrophied in the UD-E and UD-I groups (both *p* < 0.01) than in the UD-U group (which had no cases of atrophy).

**Table 1:** Patient characteristics in the group with undifferentiated-type gastric cancer according to *Helicobacter pylori* infection status

	UD-E (A)	UD-I (B)	UD-U (C)	Significance
Patients (lesions) with undifferentiated AC, n	28 (28)	32 (38)	23 (24)	
Sex				*p* = 0.001 (A vs B vs C) *p* = 0.001 (B vs C) NS (A vs B) NS (C vs A)
Male	18 (64.3%)	14 (43.8%)	20 (87.0%)	
Female	10 (35.7%)	18 (56.2%)	3 (23.0%)	
Age, years (mean ± SD)	63.1 ± 14.3	64.0 ± 13.4	56.3 ± 9.15	*p* = 0.026 (A vs B vs C) *p* = N.S (A vs B) *p* = 0.0012 (B vs C) *p* = 0.008 (C vs A)
Atrophy				*p* < 0.01 (A vs B vs C) *p* < 0.01 (B vs C) *p* < 0.01 (C vs A) *p* = 0.101 (A vs B)
None to Mild(C-0–C-2)	7 (25.0%)	3 (9.4%)	23 (100%)	
Moderate to Severe (C-3–O3)	21 (75.0%)	29 (90.6%)	0 (0%)	

AC, adenocarcinoma; NS, not statistically significant; SD, standard deviation; UD-E, post-eradication group: UD-I, current infection group; UD-U, noninfected group

### Endoscopic findings

Endoscopic findings are shown in Table 2. The lesions were located in the upper third, middle third, and lower third of the stomach in 2/9/17 cases in the UD-E group, 2/22/14 cases in the UD-I group, and 1/5/18 cases in the UD-U group, indicating that the middle third of the stomach was a significantly more common site in the UD-I group than in the UD-U group (*p* = 0.002). There was no significant difference in macroscopic type of the lesions between the groups. The color tone was reddish in about half of the cases in the UD-E and UD-I groups (42.9% and 57.9%, respectively), whereas all cases in the UD-U group were discolored (*p* < 0.001, UD-I vs UD-U; *p* < 0.001, UD-U vs UD-E).

**Table 2:** Endoscopic findings in patients with undifferentiated-type gastric cancer according to *Helicobacter pylori* infection status

	UD-E (A)	UD-I (B)	UD-U (C)	Significance
Location				*p* = 0.048 (A vs B vs C) *p* = 0.002 (B vs C) NS (A vs B, C vs A)
Upper third	2 (7.1%)	2 (5.3%)	1 (4.2%)	
Middle third	9 (32.1%)	22 (57.9%)	5 (20.8%)	
Lower third	17 (60.8%)	14 (36.8%)	18 (75.0%)	
Macroscopic appearance				NS (A vs B vs C) NS (A vs B, B vs C, C vs A)
Elevated	1 (3.6%)	2 (5.3%)	0 (0%)	
Flat or depressed	27 (96.4%)	36 (94.7%)	24 (100%)	
Color tone				p < 0.001 (A vs B vs C) *p* < 0.001 (B vs C, C vs A) NS (A vs B)
Discolored dominant	16 (57.1%)	16 (42.1%)	24 (100%)	
Reddish dominant	12 (42.9%)	22 (57.9%)	0 (0%)	

NS, not significant; UD-E, post-eradication group: UD-I, current infection group; UD-U, non-infected group

### Histopathological features

Mean tumor diameter was significantly smaller in the UD-U group (10.1 ± 5.4 mm) than in the UD-E group (19.4 ± 11.7 mm; *p* = 0.024) and UD-I group (18.6 ± 10.2 mm; *p* = 0.028). In the UD-U group, the majority of lesions (87.5%, 21/24) were pure signet ring carcinoma; this type of gastric cancer was significantly less common in the UD-E and UD-I groups (50.0% and 28.9%, respectively; both *p* < 0.01; Table 3).

**Table 3:** Histopathological findings in the patients with undifferentiated gastric cancer according to *Helicobacter pylori* infection status

	UD-E (A)	UD-I (B)	UD-U (C)	Significance
Maximum lesion diameter, mean ± SD	19.3 ± 11.7	18.6 ± 10.2	10.1 ± 5.4	*P* < 0.01 (A vs B vs C) *p* = 0.001 (C vs A) *p* = 0.005 (B vs C) NS (A vs B)
Pathological type, % of pure signet ring cell CA	50.0% (14/28)	28.9% (11/38)	87.5% (21/24)	*p* < 0.001 (A vs B vs C) *p* < 0.001 (B vs C, C vs A) NS (A vs B)
Depth, % of SM invasive cancer	14.3% (4/28)	10.5% (4/38)	0% (0/24)	NS (A vs B vs C) NS (A vs B, B vs C, C vs A)
Lymphovascular invasion, % of undifferentiated CA	10.7% (3/28)	0% (0/38)	0% (0/24)	NS (A vs B vs C) NS (A vs B, B vs C, C vs A)

CA, carcinoma; NS, not statistically significant; SD, standard deviation; SM, submucosal; UD-E, post-eradication group: UD-I, current infection group; UD-U, non-infected group

There was no significant between-group difference in the frequency of lesions with invasion into the submucosal layer or deeper (14.3%, 10.5%, and 0% in the UD-E, UD-I, and UD-U groups, respectively). All lesions in the UD-U group were intramucosal tumors. Lymphovascular invasion was seen only in the UD-E group (3 cases, 10.7%). Two of the 3 lesions with lymphovascular invasion had a tumor diameter of ≥ 30 mm. There was no significant difference compared either with the UD-I or UD-I group.

### Relationship of time since eradication with tumor diameter, color tone, and depth of invasion in the UD-E group

**Fig. 2:**
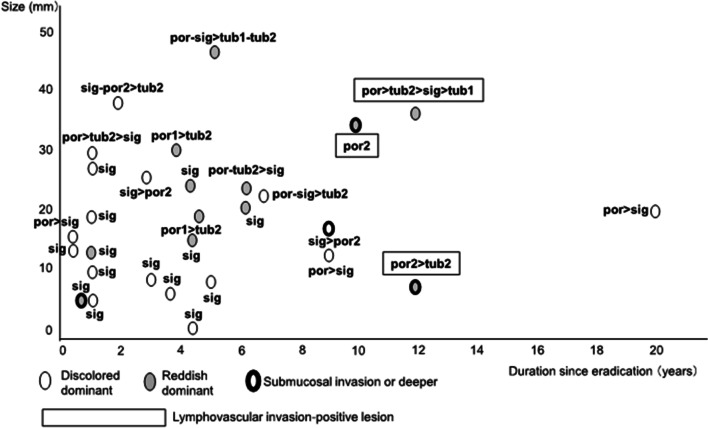
Relationship of time since eradication with tumor diameter, color tone, lymphovascular invasion, and depth of invasion in the post-eradication UD-GC cases. Por, poorly differentiated adenocarcinoma; sig, signet ring cell carcinoma; tub, tubular adenocarcinoma; UD-GC, undifferentiated-type gastric cancer

Figure 2 shows the relationship of time since eradication with tumor diameter, color tone, and depth of invasion. The median interval between eradication and detection of gastric cancer was 56 (9–240) months in the UD-E group. About 60% of UD-GCs (17 of 28 lesions) are detected within 5 years. Pure signet-ring cell adenocarcinomas are often detected early after HP eradication and tends to be smaller in size than the pathologically pure-poorly differentiated UD-GC and mixed UD-GCs. UD-GC was detected ≥ 10 years after eradication in 4 patients, 3 (75%) of whom had undergone yearly endoscopy before gastric cancer was detected. Two of the 4 lesions (50%) invaded into the submucosal layer or deeper. Three of the 4 lesions showed lymphovascular invasion.

## Discussion

The malignant potential of UD-GC is thought to be higher than that of D-GC [21]. Therefore, early detection of UD-GC is important. This study focused on clinically significant UD-GC and examined the relationship between UD-GC and HP status (post-eradication, current infection, and no infection). We found that 33.8% of all lesions in the HP-uninfected group were UD-GC, whereas only 4.5% of those in the post-eradication group and 6.2% of those in the current infection group were UD-GC. However, all tumors in the UD-U group were intramucosal lesions and not highly malignant. These cases had the following features: young age, no atrophy in the background mucosa, discolored tone, relatively small tumor size, predominantly pure signet ring cell carcinoma, intramucosal lesions, and no lymphovascular invasion. It has been reported that UD-GC without HP infection is less malignant than UD-GC with current HP infection [9, 10]. The clinicopathological features of UD-GC in patients with eradicated HP and those who were currently infected might be similar in the present study. The clinicopathological features of UD-E and UD-I are similar, including tumor size, microscopic type, color, depth, and the rate of vascular invasion, and the statistical findings are similar compared with UD-U.

Our study differs from previous reports in that we also examined the clinicopathological features of UD-GC that developed after eradication of HP. These cilinicopathological features were similar between the UD-E group and the UD-I group and showed more malignant characteristics than the UD-U group, indicating the possibility that HP infection has a role in promoting gastric cancer.

Fukase et al. reported that eradication of HP reduced the incidence of cancer to about one third [22], and there have also been studies in which eradication did not completely eliminate the risk of cancer even after a long period of time [23–26].

Alarmingly, three of four patients in whom UD-E was detected ≥ 5 years after eradication had undergone yearly follow-up endoscopy. Although this finding alone does not provide sufficient evidence to conclude that yearly follow-up endoscopy after eradication is unnecessary, endoscopists should be aware that some patients who undergo regular endoscopy may still develop cancer that is not curable by endoscopic resection.

The limitations of this study include its single-center setting and lack of surgically treated cases. Furthermore, at less than 100, the number of cases was small. Given the low frequency of UD-GC, particularly UD-E, a multicenter study is needed to accumulate and examine more cases in the future.

## Conclusion

The clinicopathological characteristics of UD-GC were similar between HP-infected patients and HP-eradicated patients. UD-GC was curable by endoscopic resection in all patients without HP infection.

## Data Availability

The datasets generated during and/or analyzed during the current study are available from the corresponding author on reasonable request. We do not wish to share data because it is identifying or confidential patient data.

## Abbreviations

HP: Helicobacter pylori
D-GC: Differentiated-type gastric cancer
UD-GC: Undifferentiated-type gastric cancer
UD-E: Undifferentiated gastric cancer after HP eradication
UD-I: Undifferentiated gastric cancer current HP infection
UD-U: Undifferentiated gastric cancer HP uninfected
